# PEPSUI, a Psychoeducational Program for the Management of Suicidal Patients: A Qualitative Study From a Randomized Controlled Trial

**DOI:** 10.3389/fpsyt.2020.500447

**Published:** 2020-09-30

**Authors:** Audrey Henrion, Philippe Courtet, Véronique Arpon-Brand, Audrey Lafrancesca, Laetitia Lacourt, Isabelle Jaussent, Sébastien Guillaume, Emilie Olié, Déborah Ducasse

**Affiliations:** ^1^CHU Montpellier, Lapeyronie Hospital, Department of Emergency Psychiatry and Post Acute Care, CHRU Montpellier, France; ^2^INSERM U1061, Neuropsychiatry: Epidemiological and Clinical Research, Montpellier, France

**Keywords:** psychoeducation, suicidal behavior, prevention, qualitative study, acceptance and commitment therapy–ACT

## Abstract

**Background:**

Suicide prevention after a recent suicide attempt remains a major issue for clinicians. Indeed, these patients are at risk of new attempts and also less prone to interact with mental health services. As psychoeducation-based interventions are strongly recommended for patients with severe or chronic disorders and poor adherence, we developed the first French program of suicide psychoeducation (PEPSUI).

**Methods:**

We started a large multicenter randomized controlled trial in outpatients who attempted suicide in the last year (i.e., current suicidal behavior disorder) to assess the feasibility, acceptability, and effectiveness of a 10-week psychoeducational program (PEPSUI group: scientific information on suicidal behavior, and third-wave cognitive behavioral therapies) compared with a 10-week relaxation program (control condition), in a naturalistic setting. Here, we present the qualitative part of this study. Participants in both groups completed a narrative interview with questions on their general impressions about the therapy process and outcomes, specific areas of change in their life since inclusion, and knowledge and perceptions about suicide and mental health services. Interviews were audiotaped, transcribed, and coded using inductive and deductive thematic analysis with a constant comparative approach. Participants were consecutively included until data saturation.

**Results:**

The interviews of 18 patients (n=10 in the PEPSUI group, and n=8 in the relaxation group) were analyzed. Qualitative analyses revealed some common points, and many differences between groups that are relevant for suicide prevention. Patients in both groups were satisfied with the programs. Group modality and therapeutic alliance with the instructors were considered useful in both groups. Participation was related to improved perception of mental health units (particularly in the PEPSUI group). Both groups reported the acquisition of stress management skills and distress tolerance. Relaxation was an easy way to survive stress. Conversely, the PEPSUI program had deeper implications for daily life through effective positioning towards internal events (thoughts and emotions) as a consequence of mindfulness-derived practices, enhancement of value-based commitments, improvement of the meaning in life and internal locus of control, increased contact with the present moment, use of a matrix (a decision-making tool), and acquisition of scientific knowledge on suicidal behavior.

**Conclusion:**

Through specific processes for targeting suicidal risk and reducing the stigma, the PEPSUI program may represent a promising intervention for suicide prevention.

## Introduction

Every year 800,000 people die by suicide worldwide and nearly 20 times more attempt suicide (1). Despite the increased effectiveness of pharmacological treatments for psychiatric diseases associated with high risk of suicide, the rates of suicidal ideation, suicide attempts, and completed suicide have not significantly decreased in recent years. The commonly accepted “stress-diathesis” model suggests that suicidal acts result from a complex interaction between vulnerability factors (diathesis) and environmental events or psychiatric diseases (stress). The most potent predictor of death by suicide is a previous suicide attempt (2), and the highest risk period for new suicide attempts is the first year following a suicide attempt (3). Moreover, suicidal behaviors may be understood as experiential avoidance strategies to reduce suffering in an increasingly addictive way (4, 5). These data highlight the need to specifically target subjects who attempted suicide in the last year (i.e., according to the DSM5 (6), individuals with current suicidal behavior disorder that might be considered a severe and debilitating disorder). Worryingly, less than half of all individuals at high suicidal risk interact with mental health services (7). One of the barriers for the management of these patients is the belief that treatment will not be effective (8).

Psychoeducation-based interventions are highly recommended for patients with severe and debilitating disorders, to increase help-seeking behaviors and to address adherence problems (9). Psychoeducational programs are effective to prevent relapse of several mental disorders, such as schizophrenia (10), bipolar disorder (11), and recurrent depressive disorder (12), and to improve treatment adherence and self-confidence in coping with the disease symptoms. The aim of psychoeducation is to propose an interactive transfer of knowledge on the disease/treatments and of management/coping cognitive/behavioral strategies, as defined by the guidelines established by the NICE (13).

Although it has been shown that educational-based preventive strategies for suicide (i.e., workshops, psychoeducational videos) improve the knowledge about suicide and positive attitudes towards help-seeking in the general population (14–16), structured psychoeducational programs for suicide prevention targeting patients at high suicide risk are still lacking. Therefore, we developed the first French psychoeducation program for suicide attempters, named PEPSUI. In accordance with the NICE guidelines (13), the aim of our program is to teach patients the most recently available knowledge about suicidal behavior and effective therapeutic strategies, through didactic and interactive group sessions. Patients are expected to become active experts in managing their disorder and in increasing adherence to treatment. Using third-wave cognitive and behavioral strategies derived from Acceptance and Commitment Therapy, Dialectical Behavior Therapy and Positive Psychology, the patients are taught to cope with unpleasant thoughts (including suicidal thoughts), unpleasant emotions (including distress), and to engage in life in a meaningful way. Third-wave cognitive and behavioral strategies have shown their feasibility, acceptability and effectiveness for the management of suicidal patients (17–19). In 2017, we started a large multicenter randomized controlled trial to assess the feasibility, acceptability, and effectiveness of PEPSUI (intervention) compared with a relaxation program (control condition), as add-on to the usual psychiatric follow-up, in outpatients with current suicidal behavior.

Here, we present the results of the qualitative part of this study that was carried out in a single center (Montpellier). We sought to collect information on the patients’ perceptions concerning the PEPSUI program and on its subjective impact in their life. Moreover, we tried to identify therapy session skills the acquisition of which the patients considered to be a significant shift and/or change in their psychological functioning, in order to extract the therapeutic processes of psychological changes. Qualitative interviews allow exploring the patients’ perspective on psychological changes, which can lead to extensive experiential information that could not be obtained by quantitative data analysis. To highlight the specificities of the PEPSUI program, we compared and contrasted the patients’ answers according to their treatment group (PEPSUI and relaxation).

## Methods

The present study was designed and carried out following the Consolidated criteria for reporting qualitative research (COREQ) (110).

### Participants

Participants were outpatients recruited from the Department of psychiatric emergency and post-acute care, Academic Hospital of Montpellier (France). Eligible participants were randomly assigned with a 1:1 ratio to follow the PEPSUI or relaxation program for 10 weeks. The randomization sequence was centralized and computed in permuted blocks of two or four by the statistician in an order unknown by the investigators. For the present qualitative study, patients were consecutively included until data saturation (20). All participants gave their written informed consent. Inclusion criteria were: age between 18 and 65 years, and current suicidal behavior disorder according to the DSM-5 (6) (i.e., suicide attempt in the last year). Suicide attempt was defined as a self-damaging act carried out with some intent to die, and distinguished from other self-destructive types of behavior, such as self-mutilation, non-compliance with medical treatment in severely ill individuals, and the use of substances such as alcohol and tobacco (21).

Exclusion criteria were: current or past diagnosis of organic mental disorder, lifetime history of schizophrenia, and mental retardation.

### Intervention Characteristics

#### Add-On Psychoeducational Program (PEPSUI; Intervention)

The PEPSUI program consisted of ten 90 min-sessions with 5 to 10 patients (1/week). Each session, was conducted by two trained instructors (nurse, medical doctor, and/or psychologist) and was focused on a specific theme or skill:

1. Education on suicidal behavior (clinic and epidemiology), and conceptualization of the phenomenon on a matrix (see **Supplementary Material**);
2. Education on the suicidal crisis, identification of important life areas and values (i.e., quality of the current behavior, how one would like to behave) for the patient, experimentation on how to use the matrix as a decision-making tool (22);
3. 3 & 4) Self-assessment of suicidal ideation, coping strategies based on suicidal ideation intensity and emotional tension (acceptation, distress tolerance, personal aid kit, and emergency care);
4. Information on the stress-diathesis model of suicidal behavior, cognitive skills (defusion), and identification of valued actions;
5. Education on stress factors (psychiatric diseases and negative life events), resilience, and learning skills to anchor in the present moment;
6. Education on suicidal vulnerability, identification of personal strengths;
7. Identification of social support, and learning skills to create quality relationships;
8. Education on treatments;
9. Conclusions.

#### Add-On Relaxation Program (Control Condition)

The intervention consisted of ten 90 min-sessions with 5 to 10 patients (1/week). Each session was conducted by two trained instructors, and focused on learning abdominal and muscle relaxation skills.

### Procedures

At the end of the 10 weeks (PEPSUI or relaxation program), a semi-structured interview was conducted to collect the patients’ subjective perspectives on the program (the interview protocol is described below). Semi-structured interviews were carried out in French, in a neutral place, by the same interviewer who was not involved in the program implementation. Participants were informed about the anonymity of their answers. All interviews lasted approximately 40 min, and were audiotaped and fully transcribed, word by word. The ethics committee of CPP Ouest II (Angers) approved the study.

### Measures

The narrative interview included 24 questions on the general impressions about the program process and outcomes (23), specific areas of change in the participants’ life since the beginning of the program, and knowledge and perceptions about suicide and mental health services.

The opening question was “What would you like to tell me about your experience with the program you just completed?”. The next questions guided the discussion on the program content and on the effect(s) on their personal functioning. First, participants were asked broad questions about their impressions of the program (e.g., “How would you describe your psychological health since the beginning of the program?”, “Was the program helpful? If yes, how?”, “Has the program improved your quality of life? If yes, how?”). Second, patients were asked more specific questions on what was useful (e.g., “As a result of the program, what changes have you done in your daily life? What do you do currently, that you never did before? If yes, how did the program make these changes possible?”, “As a result of the program, are there behaviors that you can do now, but you were not able to do before? If yes, how do you explain this?”, “As a result of the program, are there problems that you can solve now, but you were not able to solve before?”, “As a result of the program, do you think about your future differently? If yes, how?”, and “As a result of the program, has your meaning in life changed? If yes, how?”). Third, questions about the therapeutic process were asked (e.g., “Did your therapists challenge the way you thought and felt? Did your therapists challenge your private events (thoughts, emotions), perceptions, and management? Did your therapists challenge you to solve problems in a new way? How did they do this?”). Fourth, the interviewer asked questions that focused on suicidal behavior management and mental health service perception (e.g., “As a result of the program, do you think about your suicidal behavior differently? How?”, “As a result of the program, do you think about mental health services differently? How?”, and “As a result of the program, do you think that you will manage suicidal ideation differently in the future? How?”). Fifth, the interviewer probed the therapeutic relationship (e.g., “Have you ever been uncomfortable with the therapists? What did make you uncomfortable during the session(s)? How did you manage this discomfort? Have you ever been not in sync with your therapists? What happened when you and your therapists were not in sync?”). The last questions concerned the group format (e.g., «Was the group modality useful for your personal journey of change? How?»).

### Analyses

The interviews were analyzed by thematic analysis (24) with an hybrid deductive approach, based on categories *a priori* related to the theoretical framework used in the PEPSUI program, and an inductive approach (25) based on the participants’ experiences of the program. Themes were continuously compared with the data by using a constant comparative approach (26).

Several steps led this analysis (26, 27):

1. Preparation of the raw data files (data cleaning), collecting all transcripts in a common *Word* format.
2. Careful reading of the text: two raters (AH and DD), blinded to the intervention groups, read the transcripts several times to identify the most relevant themes and categories for therapeutic processes. A priori categories based on the PEPSUI framework included themes related to (a) mindfulness and emotional regulation, (b) meaning in life, (c) distress tolerance, (d) thoughts defusion, (e) relationship skills, (f) positive psychological skills, (g) matrix use, (h) suicidal behaviors perceptions, (j) mental health service perception, (k) group modality impact.
3. Creation of categories: segments of text with a specific and unique meaning were identified to create a small number of emerging categories, named by a word or a short sentence, to which meaning units were assigned (28).
4. Rating transcripts: the two raters used the codebook to rate all transcripts concerning the presence or absence of themes, and to label sections of the text that matched a category in the codebook.
5. Comparison between groups: targeted analyses identified differences between the PEPSUI group and the relaxation group; at this point, coders became aware of the group assignment. For this analysis, texts were coded and systematically compared based on the quotation types across the two groups to identify patterns. Codes were distributed among the analysis team, the texts identified with each code were read again, summaries for each code were created that included the similarities and differences across participants of the two programs. Raters then met and discussed the summaries and data audits.

## Results

Eighteen patients were included (n=10 in the PEPSUI group and n=8 in the relaxation group) to achieve data saturation (20). They were 3 men and 15 women, and their median age was 27 (min-max: 19–57) years. Nine patients were single; 11 patients lived with their spouse or with their parents, and 12 patients had no children. Eleven patients did not work.

Five main themes were identified:

1. emotional, cognitive and behavioral processes that overlapped with the following categories: a) mindfulness and openness to inner experience in an acceptance way, b) stress and distress tolerance c) defusion, d) *Self* as context (i.e., of psychological events distinct from the Self), e) values and meaning in life, f) positive affective and cognitive states, g) projection into the future, h) matrix use for effective decision making, i) new patterns of behaviors, j) improved self-awareness, and k) improved global functioning;
2. relationship-based processes that overlapped with the following categories: a) interpersonal skills in daily life, b) group format of the intervention, and c) therapeutic alliance with the instructors;
3. intervention framework;
4. suicidal behavior perception and management;
5. mental health care services’ perception.

### Similarities Between Groups

All participants in the PEPSUI group and most participants (x/8) in the relaxation group found that the intervention was helpful. Nevertheless, this help did not concern the same areas, and was not mediated by the same mechanisms. Therefore, this cannot lead to the same implications concerning suicide prevention.

All patients in the relaxation group and several patients in the PEPSUI group reported the acquisition of stress management skills and distress tolerance that are helpful to escape from a state of severe inner tension. For instance, a participant in the relaxation group said: *«We were taught to feel our alert code, which is the moment when one does not feel very well … [We were taught] to learn how to control a little bit our emotions, to ignore the whole external context»*. Then, most patients in the PEPSUI group, and some in the relaxation group described the increase of positive affects and self-esteem, and said that they changed their behavior and solved some of their problems.

Concerning suicidal behavior perception, almost all participants in the PEPSUI group, but only few patients in the relaxation group described changes in their perceptions and thoughts about the future management of suicidal thoughts.

A common useful process in both groups was the confident relationship between patients and instructors. The instructors’ availability and empathic listening were considered to be a very supportive factor.

The group format was helpful for most patients in both groups. The group was perceived as a space for sharing experiences in a supportive and non-judgmental way. Patients said that they felt no longer alone in front of the disease. They found that the group format made easier the implementation of the therapeutic exercises. A PEPSUI participant reported *“As we were in a small group, we had the opportunity to talk, to know a little about the worries of the others … we were not alone … We all had to say something and it was good to do it with people who were listening to us and who were not necessarily in the medical field.”* Participants in both groups also emphasized the benefits of the weekly schedule and their active involvement as a motivation to change.

Finally, more than half of patients in the PEPSUI group, and less than half of patients in the relaxation group reported a change in their perception of mental health services as a result of the program: decreased stigma and improved attitudes towards help-seeking in such units.

### Differences Between Groups

Although there were similarities between groups, the differences were more important.

All patients in the PEPSUI group (but none in the relaxation group) reported improved emotional regulation, through mindfulness skills. They mentioned the anchor in the present moment as a moderator of unpleasant inner feelings, particularly proneness to anger, sadness, and hopelessness, and also social adversity feelings. All these dimensions are involved in suicidal vulnerability. A patient explained “*It allows focusing on us, on what is currently disturbing us, what is the current emotion going on in us … [it allows us to know] what is the heart of the problem, and how to handle it*”. Patients in the PEPSUI group also associated anchor in the present moment with decreased rumination. Furthermore, PEPSUI patients linked acceptance and openness to the ongoing experiences to improved emotional regulation. For example, a patient said: “*Before the therapy, I was feeling so bad that I told myself that I was going to kill myself … I was feeling so bad that I couldn’t manage anything … It was unbearable … Therefore, I told myself that I couldn’t live like that. I was the victim of my emotions that were causing physical problems. It became unbearable. But now, I do not have that anymore. I can regulate my emotions, I can understand what I feel, accept that we can have unpleasant sensations and that this will always happen*”. The process of emotion acceptance was described by patients as a precise inner investigation of bodily sensations, in order to define the real place and modalities of ongoing emotional sensations. Emotional experience takes no longer all the space in one’s experience, but is rather limited to a precise place in the body. Here, are examples of how patients described this change: “*They taught us to perform mindfulness practices, that is to anchor in life here and now … to be aware of the current pain, whether it is a lump in the abdomen, a tension or a kind of warmth in the neck … to be aware of the pain as we feel it … and now it does not take all the importance I gave to it at the beginning, I can accept this pain*”, “*I can accept this pain and tell myself: OK, it is just a pain, it is not something that must take over all my time or all my body … it does not take the whole place and I can resume my daily life … it is a pain, but it is not something that has to take the whole place to the point of attempting suicide, whereas it was the case before the program”, “I accept to experience emotions. Before the program, I transformed [emotions] all the time. As soon as I was afraid, anxious or ashamed, I used to transform these emotions into anger. Now I accept emotions as they are, I do no longer transform them”, “When we feel something unpleasant, [we need to] accept it, not to struggle in order not to feel it because otherwise it’s worse”*. Some patients in the relaxation group reported an improvement in stress management, but they were not able to explain the underlying psychological processes, or attempted to suppress unpleasant experiences (i.e., experiential avoidance). They used relaxation skills to escape from the ongoing stress in a short-term manner. Some patients in the PEPSUI group also explained that they tried to suppress unpleasant thoughts and emotions, but this led to an increase of their intensity. This suggests that the psychotherapeutic processes are fundamentally different in the two groups, as highlighted by the following statements. A participant in the relaxation group said: “*It [relaxation] acts at least as a bit of a distraction, it allows us to avoid thinking about our memories that generate anxiety or about our expectations*”. A participant in the PEPSUI group said: “*It is not a question of thinking to something else because it is there, it exists, so we cannot actually deny it … I have learned to be aware of this pain that should not take all the place”*. Concerning thoughts, patients in the PEPSUI group described a defusion process: “*They taught us that a thought was just a thought, we had to let it come and not to struggle against it … Thoughts arise, it is independent from us, it is a normal thing…”*. Patients in the PEPSUI group described a modification in their attitude towards mental events (thoughts and emotions) that had an impact on their perception of the daily life experiences. *“I am completely different from when I started the program. It is not necessarily about [external] changes, but rather about my way of understanding things … I have now a different point of view compared to the beginning of the program”, “This program is a revelation of myself … an awareness about myself, a perspective from my emotions”*. The majority of patients in the PEPSUI group reported that they modified their mental events’ perception. Conversely, few patients in the relaxation group described modifications of their inner experience based on the learned stress management skills. Patients in the PEPSUI group focused their statements on skill acquisition and examples from the instructors, whereas patients in the relaxation group focused their statements on the therapeutic alliance. For instance, participants in the PEPSUI group highlighted the importance of the instructor’s self-confessions: *“She [i.e., the instructor] explained the difficulties she had encountered in her life … we recognized ourselves in her … the fact that she understands what we live and she shares it with us can only comfort us. We told to ourselves that if she has managed to change things for her, we should also be able to do the same.”, “[Instructors] are not here to demonstrate things they have read in books … During the sessions, I have understood that they applied [the principles of the program] to themselves too … They [i.e., the instructors] are individuals like us who use therapy and its tools to better manage their daily lives”*.

Many patients in the PEPSUI group reported that skills derived from the matrix utilization were an effective way to analyze and cope with issues. The matrix use, leading to “*another angle of view*”, was recalled by patients as follows: “*It is very helpful. Now, as soon as there is something that is problematic for me, I make a matrix. In this way, I do not make mistakes*”, “*When my emotions are overwhelming*, *I make [write down] a matrix in order not to act impulsively … And then, in the long term, I will not need to make it anymore because it will become automatic*”*, “It has helped me to become aware, to think about how to do things and to find solutions”*. All the patients in the PEPSUI group reported a positive change of their mental health, and almost all an improved quality of life. They reported to solve problems and to have implemented relevant changes in their daily lives. Conversely, in the relaxation group, only four patients (50%) mentioned an improvement in their mental health and quality of life related to better stress management. Few patients in the relaxation group reported changes in their life, mostly by using stress management skills. “*Every day, at home or outside, when I am in my car or in the street, I practice belly breathing*”. Among the changes in their daily life described by patients in the PEPSUI group there was the development of relevant meaningful actions to improve self-determination in life. “*Only I can be the person who will manage my life in a positive way … one step at a time, I am getting closer to the person I really want to be and I am moving away from my vicious circle”*. Patients in the PEPSUI group also reported the acquisition of skills in interpersonal relationships, leading to a better management of conflicts and increased self-esteem. *“[Before the program] an argument with my partner took all the space, and I was unable to quietly talk about this subject the day after … Now, my relationships with others are more serene”*. Patients in the PEPSUI group said that they developed social bonds thanks to several processes: contact with the present moment, improved emotional regulation, analysis of a given situation through the lens of the matrix. They also reported the importance given to openness, generosity and contribution to others: “*I felt the urge to help others, to help with my means”, “We must not wait to receive, we must give. [We must] realize that finally we all have things to give … No matter what we give, we can be useful for something”*. Patients thought that they finally found a social place: *“We end up taking our place in society or in the couple, we gain self-confidence”*. Few patients in the relaxation group reported improvements in their interpersonal skills. Concerning the projection into the future, it was strongly broadened with increased optimism in patients from the PEPSUI group, but not from the relaxation group. A patient in the PEPSUI group commented “*I can imagine a positive, healthy and stable life, whereas before [the program] it wasn’t the case at all. I have a different vision compared to when I began the program”*.

Perception of suicidal behaviors, at the core of the PEPSUI program, differed between groups. Patients in the PEPSUI group highlighted that they perceived differently suicidal ideation/act compared with before the program. They also felt that their ability to manage future suicidal ideation was improved. This was related to: 1) the understanding of how suicidal ideation emerges using the matrix, 2) the acquisition of acceptance skills, leading to an effective emotional regulation, 3) the decrease of guilt related to the previous suicide attempt, and 4) the decrease of self-stigma through acceptance of their mental disorder. “*In general, we arrive to the suicidal act to get relief from a pain, or to decrease it. If we learn how to manage pain, we know that it has a peak, and that the peak will go away, and it will not be there for our entire life. At the beginning, I thought that when there was a suicidal behavior, it was because we told ourselves that nothing could change, that it would be the same at vitam eternam … Finally, we are aware that we will have to endure it for only few minutes, or few hours at most; [we are aware that] after, it will cease, and therefore we just have to keep pace.”, “They explained to us that many people try to commit suicide … [contributing] to not feel alone in the world … we realized that there are many people in our situation … I needed to understand what led me to attempt suicide”*. Several patients in the PEPSUI group explained how the struggle against mental experiences increased suicidal ideation, sometimes leading to a suicidal act. Half of the participants in the PEPSUI group spontaneously said that scientific knowledge on suicidal behaviors was helpful. *“To learn about the genetic components decreased my culpability, because I told myself that it didn’t come from me, that it wasn’t entirely my fault”*. In the relaxation group, only one patient reported a modified perception of suicidal behaviors, and four patients found that stress management had an impact on their ability to face suicidal ideation. Several patients in the PEPSUI group, but none in the relaxation group, reported decreased frequency of non-suicidal self-harm through improvement of emotional regulation. *“My happiness is expanding on a daily basis. When there is an argument with my boyfriend, I no longer bang my head against the walls, there is no more self-injurious behaviors at all”, “[Before the program] I did a lot of scarification … since [the program], I feel a lower need for it”*. Finally, patients in the PEPSUI group said that they were more prone to develop help-seeking behaviors: *“It will be easier for me [than before the program] to contact someone rather than to attempt suicide. Before [the program] I would not have dared to go to the psychiatric emergency department or to call the emergency mobile unit. But now I could do it more easily if something unpleasant were arising for me that I couldn’t cope with”*.

## Discussion

This is the first qualitative study assessing psychological changes and the underlying processes reported by outpatients at high-risk of suicide enrolled in a psychoeducational (PEPSUI) or relaxation program. Our results highlight the skills that are relevant for suicide prevention, particularly those specifically developed in the PEPSUI integrative program based on third-wave cognitive/behavioral strategies.

Patients were satisfied with the quality of care independently of the group to which they were allocated. They reported improved perception of mental health services, decreased self-stigma, and improved attitudes towards help-seeking. In agreement with the scientific literature (29), group modality and therapeutic alliance with the instructors were considered useful by all participants. However, the therapeutic alliance process was different in the PEPSUI and relaxation groups. Indeed, in the relaxation group, it was related to the empathic instructor’s presence, whereas in the PEPSUI group, it was related to the transmission of new skills with examples through self-confessions by the instructor. The aim of such self-confessions was to foster a spirit of collaboration with the patients on tasks, in a human-to-human relationship. It rooted the transdiagnostic collaborative suicide-specific framework for alliance-building, which has shown its interest for suicidal management (30). In both groups, patients reported the acquisition of stress management skills and distress tolerance. The relaxation program focused on stress management (as an end in itself). Conversely, the PEPSUI program considered such strategies as an emergency plan to manage high levels of inner tension, but only when emotional regulation strategies failed. Relaxation appeared as a way to survive stress, whereas the PEPSUI program involved specific psychological processes to act on the daily life.

Patients in the PEPSUI group reported modifications in their attitudes towards internal experiences (i.e., thoughts and emotions) thanks to mindfulness-derived practices that were based on openness to the current experiences, thought defusion, and emotion acceptance. This is in line with previous findings showing the positive effect of mindfulness skills on suicidal ideation (31). Specifically, patients experientially understood that attempting to suppress unpleasant thoughts (including suicidal ideation) is counterproductive. It has been shown that experiential avoidance is associated with increased intensity and frequency of unpleasant psychological events, including suicidal ideation (32–35). Interestingly, patients in the PEPSUI group reported that they felt able to manage suicidal ideation in a more effective way than before. Patients understood that unpleasant emotions are expressed through bodily sensations that induce psychological pain (24), leading to the urgent need to act and possibly to adopt experiential avoidance behaviors (25). Indeed, suicidal behavior may be considered as an extreme experiential avoidance strategy to escape from intolerable psychological pain. Moreover, acceptance skills decrease pain catastrophizing (i.e., the tendency to magnify or exaggerate the threat value or seriousness of pain sensations) (36) that has been associated with increased suicidal ideation/act in patients with headache (37).

Patients in the PEPSUI group specifically reported modifications in their involvement in various daily life areas. Valued commitments were sought for their own sake, just for the pleasure of doing them. This kind of commitments have been related to the “optimal experience” or “flow”, described by Mihály Csíkszentmihályi (38). The optimal experience is not something that passively happens, depending on pleasant external conditions, but rather something that actively happens depending on one’s involvement in life (whatever the external conditions) in a valued-based-state of mind. It is related to intrinsic motivation, i.e., the ability to find enjoyment and purpose regardless of the external circumstances (39). In the long term, valued commitments enhance the sense of participation in determining the content of life. People often try to achieve life satisfaction by pleasure maximization (28), leading to increased vulnerability due to the way impermanent external reality occurs. In suicidal patients, motivation to hedonic experiences is reduced, pain avoidance is increased (40), and sense of purpose in life is decreased (41). Contact with values may promote the internal locus of control (42), thus decreasing suicide risk (43). Patients in the PEPSUI group reported improved self-esteem, and quality of the relationships with others. According to the interpersonal theory of suicide (44), this may have an impact on suicidal ideation. This theory proposes that suicidal ideation occurs when subjects experience low belongingness (i.e., social disconnection) and high burdensomeness (i.e., to be a burden to others). Patients in the PEPSUI group reported that they shifted their attention from worries about their impaired Self, to benefitting others, which is deemed to foster mental health (45). Patients’ statements echo Viktor Frankl’s statement in *Man’s Search for Meaning* (46): “Happiness cannot be pursued; it must ensue … as the unintended side-effect of one’s personal dedication to a course greater than oneself”. Literature data indicate that low meaning in life is associated with depression and suicide (47), whereas high meaning in life protects against suicidal ideation (41, 48). Participants in the PEPSUI group reported contact with the present moment, defined as being fully involved with every detail of their life. It leads to the capacity to appreciate what is already here, and to the awareness of all people involved in a single moment of life. This is intrinsically related to gratitude skills that are useful in suicidal patients (19). In addition, contact with the present moment favors the involvement in valued-based commitments. Usually, people are too focused on what they want to achieve and therefore, they fail to enjoy the present moment. Valued commitment involves deriving contentment from the action for its own sake, in the present moment. Finally, patients in the PEPSUI group linked better contact with the present moment to decreased rumination, which has been implicated in suicidal behaviors (49).

Patients in the PEPSUI group found that the matrix (22) was an effective decision-making tool to analyze and to cope with difficult situations, and to decrease impulsivity. This visual tool seeks to increase psychological flexibility (50), leading to the ability to choose valued actions in the presence of unpleasant psychological events. Therefore, the PEPSUI program may have an effect on neuropsychological features of suicidal risk: impaired decision making (51), reduced cognitive flexibility (52), and poor problem-solving abilities (53).

In the PEPSUI group, scientific knowledge about suicidal behavior contributed to understanding that suicidal ideation is the outcome of interactions among many factors, including stable biological factors. This understanding was linked by the patients to a decreased culpability towards their suicidal experiences that were understood as part of a mental disorder affecting many people. Patients in the PEPSUI group said that they learnt to accept their mental disorder.

This study has some limitations. First, all the consecutively included patients in the PEPSUI group (n=10) could be interviewed. Conversely, 14 patients had to be included in the relaxation group to reach the number of patients needed for reaching data saturation (n=8) (37), because 6 patients were lost of follow-up. This could suggest that PEPSUI was perceived as more useful than relaxation. Second, in line with the recommendations on qualitative studies (20), the number of included subjects (n=18) was enough to achieve data saturation and to draw conclusions. However, our findings cannot be generalized. Third, the instructors in the relaxation group attended mindfulness meditation sessions each week. We cannot exclude that the relaxation instructors were influenced by the mindfulness skills they integrated in their own life. Fourth, the group was an add-on program in a naturalistic setting. Therefore, the concomitant care may have affected the patients’ perceptions of the PEPSUI and relaxation programs. Last, the qualitative assessment was performed only at the end of the program. Future studies with randomized samples should include multiple longitudinal qualitative assessments to draw a broader picture of the impact of psychoeducational therapy, particularly on the long-term mechanisms at work, and to identify subgroups of patients who are more likely to benefit from the program.

This study has several strengths. First, it relied on a robust methodology because we used a randomized controlled design. Due to the limited exclusion criteria, a representative suicidal outpatients sample was included. The methodology design was well adapted to identify specific and non-specific psychological processes involved in PEPSUI. The qualitative methodology was the most appropriated to explore the internal subjective experiences and processes, which could not have been done with quantitative methods. Semi-structured interviews allowed respecting the spontaneous input from patients, while keeping a neutral attitude. The semi-structured interviews were carried out in a neutral place, by the same interviewer who was not involved in the groups’ animation. The content validity was respected using a literature-based guide for narrative interviews. To prevent potential bias resulting from different meanings that interviewers and interviewees could attribute to words, reformulations and summaries of the patients’ input were performed throughout the interviews. Furthermore, verbatim transcriptions were rigorous and hand-written to avoid inaccuracies from computer programs. The present study fitted the Consolidated criteria for reporting qualitative research (COREQ) criteria (110), and the saturation data principle was respected (80). Finally, a qualitative study of a psychoeducational program for suicide prevention is innovative.

## Conclusion

To overcome the gap between the need and the availability of evidence-based treatments, cost-effective low-threshold accessible interventions must be developed. Our qualitative study indicates that the PEPSUI psychoeducational program may represent a promising intervention for suicide prevention. According to the patients’ opinion, the PEPSUI program was a way to expand and develop a meaningful life, whereas relaxation appeared only as a way to survive stress.

## Data Availability Statement

All datasets generated for this study are included in the article/**Supplementary Material**.

## Ethics Statement

The studies involving human participants were reviewed and approved by the ethics committee from the CPP Ouest II (Angers) approved the study. The patients/participants provided their written informed consent to participate in this study. Written informed consent was obtained from the individual(s) for the publication of any potentially identifiable images or data included in this article.

## Author Contributions

All authors contributed to the article and approved the submitted version.

## Conflict of Interest

The authors declare that the research was conducted in the absence of any commercial or financial relationships that could be construed as a potential conflict of interest.
