# Supplementary material for: PEPSUI, a Psychoeducational Program for the Management of Suicidal Patients: A Qualitative Study From a Randomized Controlled Trial
Source: Front Psychiatry. 2020 Sep 30;11:500447. doi: 10.3389/fpsyt.2020.500447 (PMC7561374; doi:10.3389/fpsyt.2020.500447)
Supplement: Supplementary file 1 [file DataSheet_1.docx]

Supplementary material. The ACT matrix

The Matrix is a simple method for teaching people to understand the function of their behaviors (e.g., avoidance/escape from mental suffering versus moving toward values) as well as to discriminate between internal or external events (e.g., mental experiencing and direct experience). It was created by Kevin Polk, Mark Webster, and Jerold Hambright.

| Outside | | | |
| --- | --- | --- | --- |
| Away | What do you do to deal with, to control, or to move away from inner experience you don't want to have ? | Behaviours that move you toward what matters - while having the unwanted inner experience | Toward |
|  | What inner experience gets in the way of moving toward ?  What have you been moving away from ? | Who is important to you?  What is important to you? |  |
| Inside | | | |
